# Supplementary material for: Thermal modulation of Zebrafish exploratory statistics reveals constraints on individual behavioral variability
Source: BMC Biol. 2021 Sep 21;19:208. doi: 10.1186/s12915-021-01126-w (PMC8456632; doi:10.1186/s12915-021-01126-w)
Supplement: Supplementary file 1 — Additional file 1 Figure S1: Correlations between parameters. A Pearson’s correlation coefficients between per-bout parameters, reorientation angles of turn bouts, interbout interval and displacement. B Pearson’s correlation matrix between temperature-averaged parameters. C Variance explained by the principal components of the inter-temperature matrix. D Eigenvalues of the pooled intra-temperature matrix. The red line highlights the Kaiser-Guttman criterion. [file 12915_2021_1126_MOESM1_ESM.pdf]

Additional file 1

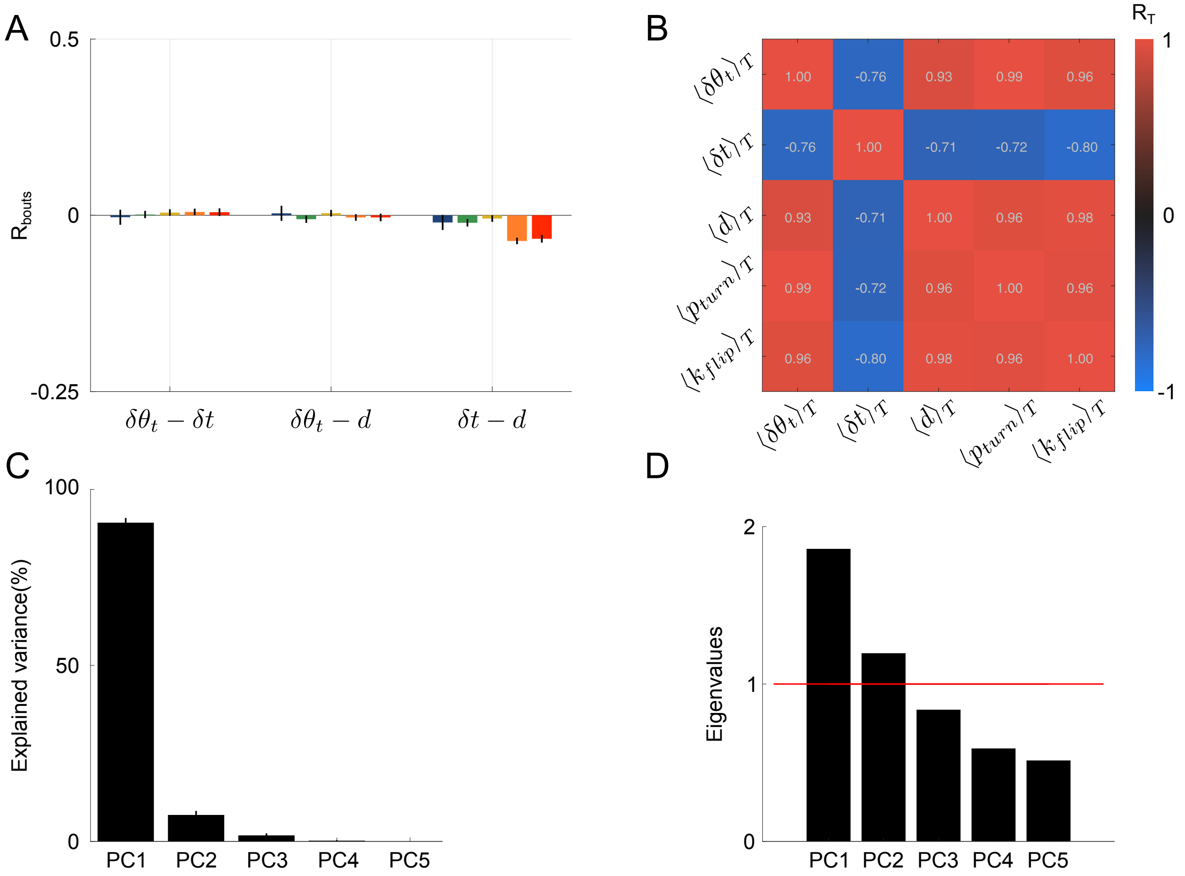

Figure S1: Correlations between parameters. **A** Pearson's correlation coefficients between per-bout parameters, reorientation angles of turn bouts, interbout interval and displacement. **B** Pearson's correlation matrix between temperature-averaged parameters. **C** Variance explained by the principal components of the inter-temperature matrix. **D** Eigenvalues of the pooled intra-temperature matrix. The red line highlights the Kaiser-Guttman criterion.
