# Supplementary material for: Thermal modulation of Zebrafish exploratory statistics reveals constraints on individual behavioral variability
Source: BMC Biol. 2021 Sep 21;19:208. doi: 10.1186/s12915-021-01126-w (PMC8456632; doi:10.1186/s12915-021-01126-w)
Supplement: Supplementary file 2 — Additional file 2 Figure S2: PCA in single-fish experiments. A Variance explained by the five principal components for each single-fish. B-C Autocorrelation function of the projection on PC1 (B) and PC2 (C) from each fish in single-fish experiments. The color code is the same as in A, black line and shaded area is the mean and s.e.m. across fish. D Mean variance of projections across time (intra, purple) and overall variance of projections (green). Error bars for intra is the s.e.m. and error bars for overall is 95 confidence intervals after bootstrapping (n=1000 boots). [file 12915_2021_1126_MOESM2_ESM.pdf]

Additional file 2

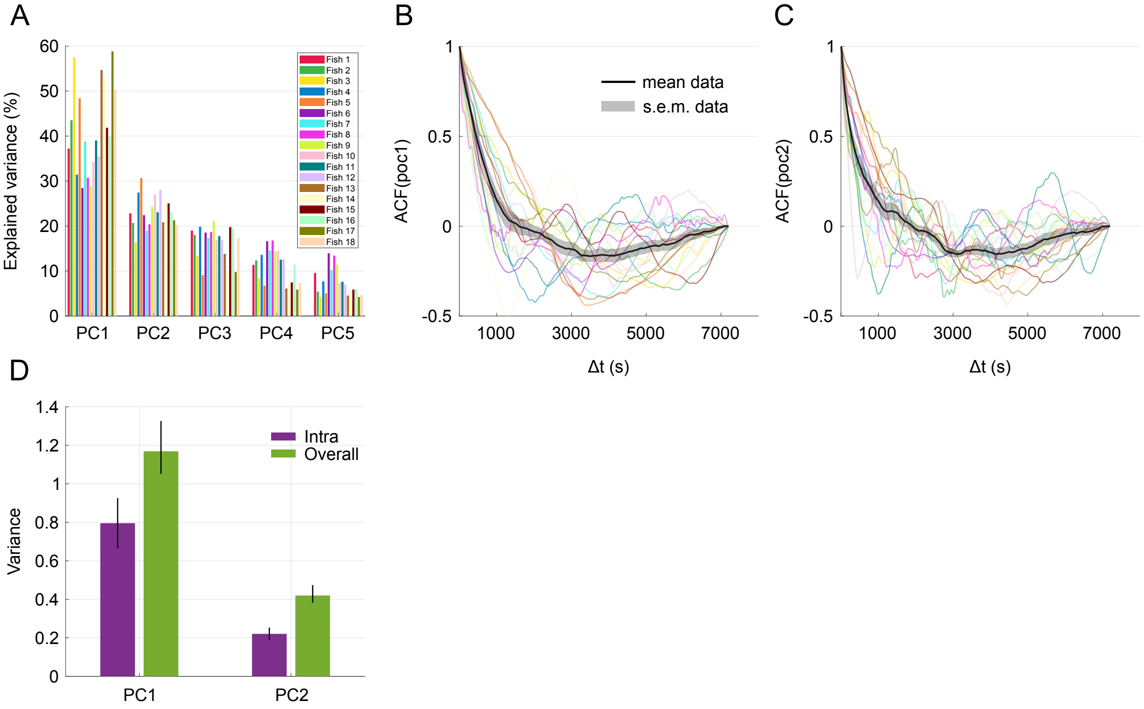

Figure S2: PCA in single-fish experiments. **A** Variance explained by the five principal components for each single-fish. **B-C** Autocorrelation function of the projection on PC1 (B) and PC2 (C) from each fish in single-fish experiments. The color code is the same as in A, black line and shaded area is the mean and s.e.m. across fish. **D** Mean variance of projections across time (intra, purple) and overall variance of projections (green). Error bars for intra is the s.e.m. and error bars for overall is 95% confidence intervals after bootstrapping (n=1000 boots).
