# Supplementary material for: Thermal modulation of Zebrafish exploratory statistics reveals constraints on individual behavioral variability
Source: BMC Biol. 2021 Sep 21;19:208. doi: 10.1186/s12915-021-01126-w (PMC8456632; doi:10.1186/s12915-021-01126-w)
Supplement: Supplementary file 3 — Additional file 3 Figure S3: Temperature-independant rescaling of parameters. A Equation describing parameter X distribution. B-E Left to right, temperature-averaged value, trajectory-averaged rescaled by temperature averaged-value and per-bout value rescaled by the trajectory average, for B interbout intervals, C displacements, D reorientation angle of turn events, E turning probability. [file 12915_2021_1126_MOESM3_ESM.pdf]

Additional file 3

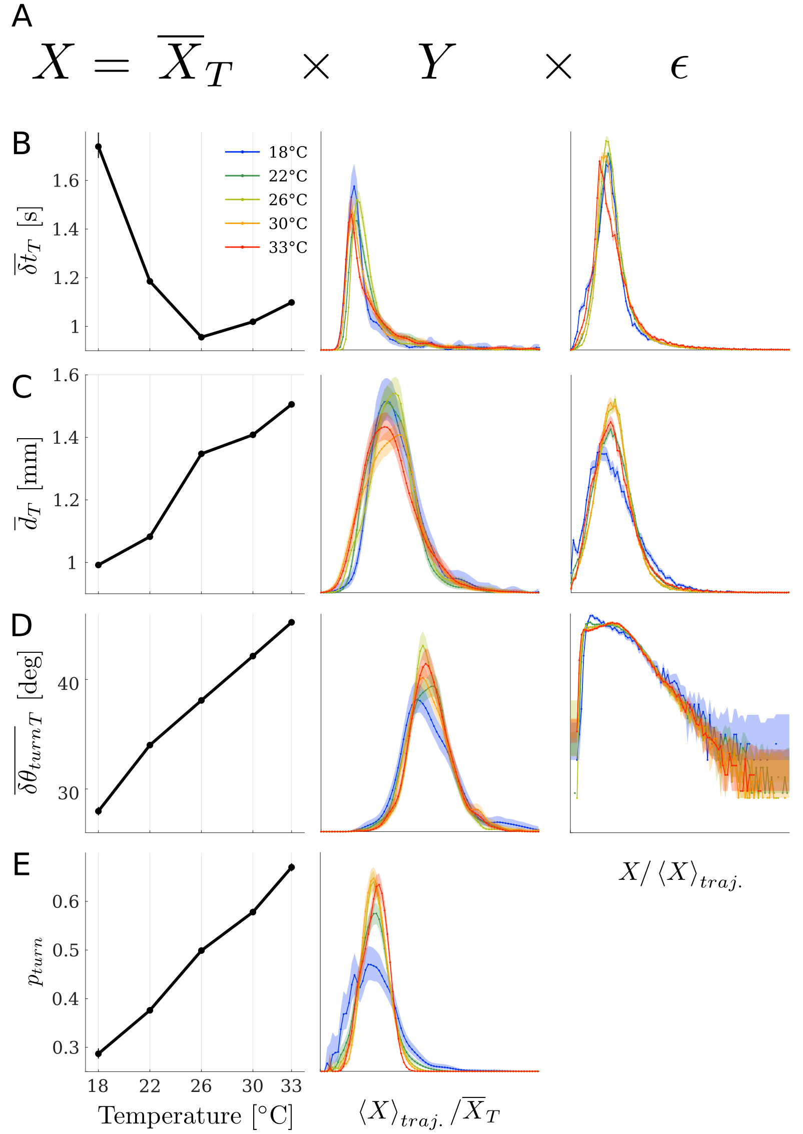

Figure S3: Temperature-independant rescaling of parameters. **A** Equation describing parameter X distribution. **B-E** Left to right, temperature-averaged value, trajectory-averaged rescaled by temperature averaged-value and per-bout value rescaled by the trajectory average, for **B** interbout intervals, **C** displacements, **D** reorientation angle of turn events, **E** turning probability.
