# Supplementary material for: Thermal modulation of Zebrafish exploratory statistics reveals constraints on individual behavioral variability
Source: BMC Biol. 2021 Sep 21;19:208. doi: 10.1186/s12915-021-01126-w (PMC8456632; doi:10.1186/s12915-021-01126-w)
Supplement: Supplementary file 4 — Additional file 4 Figure S4: Fish position distributions along a linear thermal gradient. Presence probability density function of 10 batches of 10 larvae experiencing a thermal gradient from 18 ∘C to 33 ∘C. Solid line is the mean across batches, shaded area is the s.e.m. Dashed line is the expected value for a uniform distribution. [file 12915_2021_1126_MOESM4_ESM.pdf]

Additional file 4

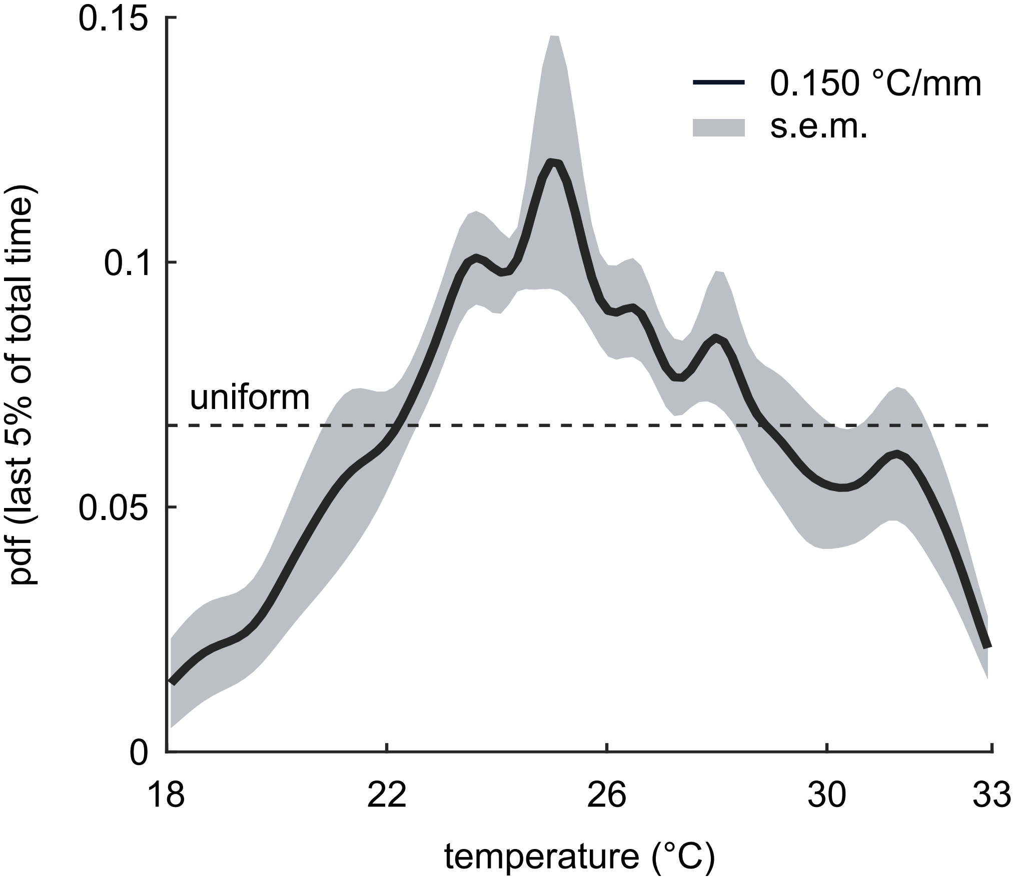

Figure S4: Fish position distributions along a linear thermal gradient. Presence probability density function of 10 batches of 10 larvae experiencing a thermal gradient from 18°C to 33°C. Solid line is the mean across batches, shaded area is the s.e.m. Dashed line is the expected value for a uniform distribution.
