# Supplementary material for: Thermal modulation of Zebrafish exploratory statistics reveals constraints on individual behavioral variability
Source: BMC Biol. 2021 Sep 21;19:208. doi: 10.1186/s12915-021-01126-w (PMC8456632; doi:10.1186/s12915-021-01126-w)
Supplement: Supplementary file 5 — Additional file 5 Figure S5: Controls. A Example statistics of three batches successively recorded in E3 (control) and in E3 previously heated at 45 ∘C for one hour in the experimental setup. (Left to right, up to bottom) Mean interbout interval, mean displacement, amplitude of turn bouts reorientation angle and mean square reorientation. B Example statistics from all experiments pooled together, dividing time into three windows of 10 min. (Left to right, up to bottom) Mean interbout interval, mean displacement, amplitude of turn bouts reorientation angle. C Example statistics at a given temperature (current T) as a function of the previously tested temperature. One dot is the mean parameter value for one experiment, color encoded current temperatures, lines are the mean for each previous temperature. (Left to right, up to bottom) Mean interbout interval, mean displacement, amplitude of turn bouts reorientation angle. [file 12915_2021_1126_MOESM5_ESM.pdf]

Additional file 5

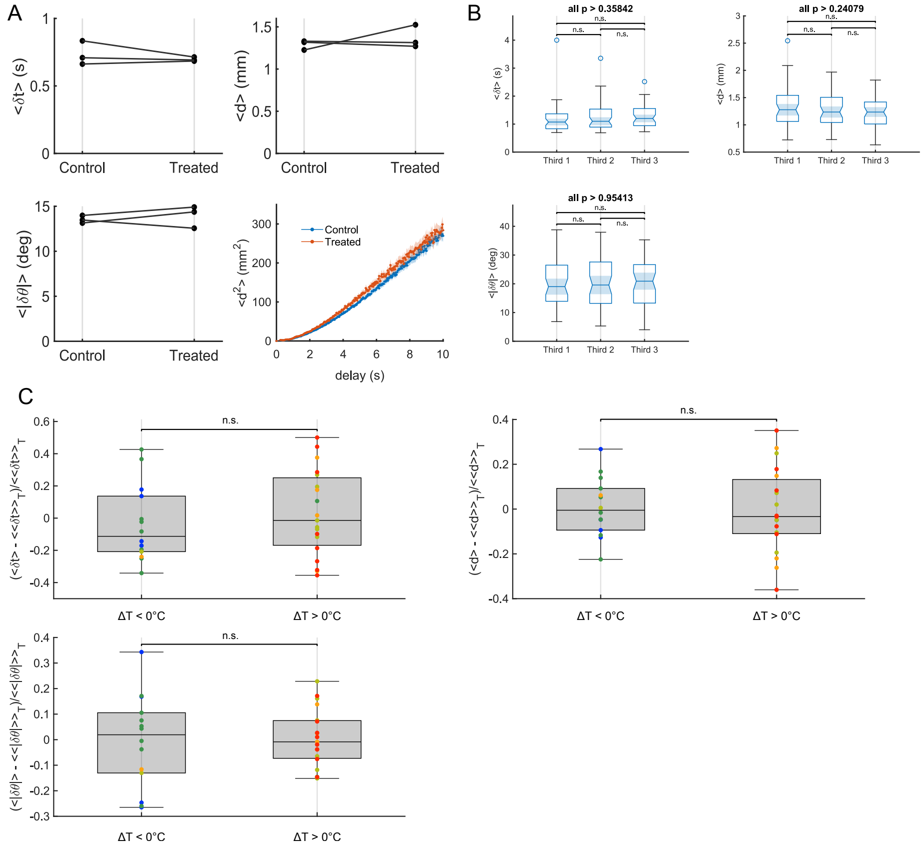

Figure S5: Controls. **A** Example statistics of three batches successively recorded in E3 (control) and in E3 previously heated at 45°C for one hour in the experimental setup. (Left to right, up to bottom) Mean interbout interval, mean displacement, amplitude of turn bouts reorientation angle and mean square reorientation. **B** Example statistics from all experiments pooled together, dividing time into three windows of 10 minutes. (Left to right, up to bottom) Mean interbout interval, mean displacement, amplitude of turn bouts reorientation angle. **C** Example statistics at a given temperature (current T) as a function of the previously tested temperature. One dot is the mean parameter value for one experiment, color encoded current temperatures, lines are the mean for each previous temperature. (Left to right, up to bottom) Mean interbout interval, mean displacement, amplitude of turn bouts reorientation angle.
