# Supplementary material for: Thermal modulation of Zebrafish exploratory statistics reveals constraints on individual behavioral variability
Source: BMC Biol. 2021 Sep 21;19:208. doi: 10.1186/s12915-021-01126-w (PMC8456632; doi:10.1186/s12915-021-01126-w)
Supplement: Supplementary file 6 — Additional file 6 Figure S6: Effect of cut-offs in trajectory selection. A-B Example statistics when changing cutoffs in trajectory selection. (Left to right, up to bottom) Mean interbout interval, mean displacement, fraction of turns, amplitude of turn bouts reorientation angle, first principal component coefficients, second principal component coefficients. A With a minimum time between two consecutive bout of 200ms, trajectory must last at least 25 s. B With a minimum time between two consecutive bout of 400ms, trajectory must last at least 5 s. [file 12915_2021_1126_MOESM6_ESM.pdf]

Additional file 6

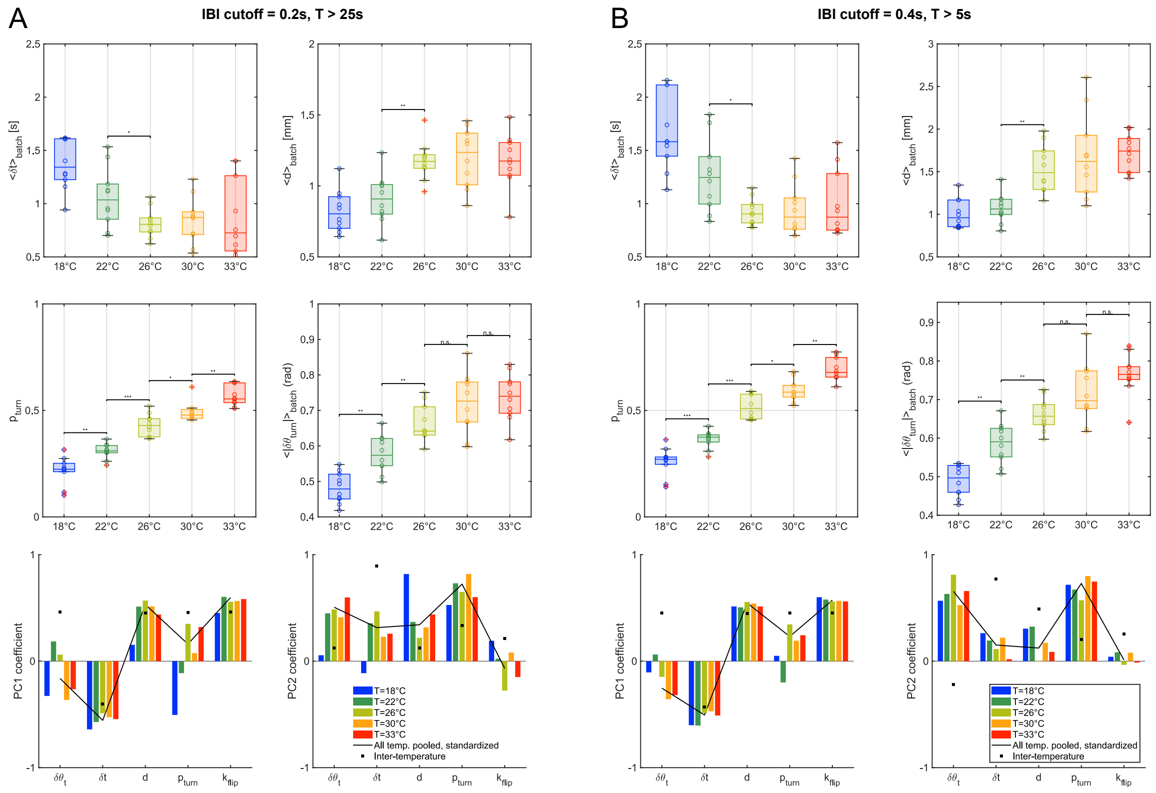

Figure S6: Effect of cut-offs in trajectory selection. **A-B** Example statistics when changing cutoffs in trajectory selection. (Left to right, up to bottom) Mean interbout interval, mean displacement, fraction of turns, amplitude of turn bouts reorientation angle, first principal component coefficients, second principal component coefficients. **A** With a minimum time between two consecutive bout of 200ms, trajectory must last at least 25 seconds. **B** With a minimum time between two consecutive bout of 400ms, trajectory must last at least 5 seconds.
